# Multi-Modal Data-Driven Bayesian-Optimized CNN-LSTM Model for Slope Displacement Prediction

**DOI:** 10.3390/s26051452

**Published:** 2026-02-26

**Authors:** Xingwang Zhao, Xinlong Wan, Jian Chen, Chao Liu, Chao Chen

**Affiliations:** 1 Key Laboratory of Aviation-Aerospace-Ground Cooperative Monitoring and Early Warning of Coal Mining-Induced Disasters of Anhui Higher Education Institutes, Anhui University of Science and Technology, Huainan 232001, China; 2 State Key Laboratory for Safe Mining of Deep Coal Resources and Environment Protection, Anhui University of Science and Technology, Huainan 232001, China; 3 School of Spatial Information and Geomatics Engineering, Anhui University of Science and Technology, Huainan 232001, China

**Keywords:** slope displacement, multi-modal data, Bayesian optimization algorithm, CNN, LSTM

## Abstract

**Highlights:**

**What are the main findings?**

- A multimodal data-driven Bayesian optimized CNN-LSTM prediction model was constructed, which significantly improved the accuracy and stability of slope displacement time series prediction.
- The study verified that fusing multimodal data such as rainfall and earth pressure can effectively enhance the model’s ability to represent external influencing factors, thereby improving prediction stability.

**What are the implications of the main findings?**

- This provides a high-precision intelligent prediction method for slope safety monitoring and geological disaster early warning, supporting reliable extrapolation prediction in the case of missing or abnormal GNSS data.
- The constructed framework provides a technical approach that can be referenced for similar engineering time series prediction tasks.

**Abstract:**

Accurate prediction of slope displacement is an important prerequisite for building an effective geological hazard early warning system for disaster prevention and reduction. However, the inherent nonlinearity and time-varying characteristics of slope displacement evolution greatly affect the prediction accuracy. To improve the slope displacement prediction accuracy, a multi-modal data-driven Bayesian-optimized Convolutional Neural Network and Long Short-Term Memory (Bayes-CNN-LSTM) model was constructed. The performance of the model was evaluated using multi-modal monitoring data from the GuShan mine slope. Experimental results showed that the Bayes-CNN-LSTM model achieved an average coefficient of determination (R^2^) of 0.971, with a mean absolute error (MAE) of 0.444 mm and a root mean square error (RMSE) of 0.618 mm. Compared with the CNN-LSTM, LSTM, CNN, SVM, TCN, and Transformer models, the MAE of the constructed model was decreased by 25.1%, 31.3%, 32.3%, 24.1%, 24.7%, and 17.7%, respectively, and the RMSE decreased by 20.1%, 26.9%, 29.5%, 18.0%, 20.7%, and 12.4%, respectively. Furthermore, the proper integration of multi-modal data can effectively improve the prediction accuracy when extrapolating slope displacement. Based on rainfall and earth pressure data, the average MAE and RMSE of extrapolation (24-h) prediction using the constructed model were decreased by 30.2% and 24.6%, respectively. The model effectively improves the accuracy of slope displacement prediction and enhances the practicality of the slope safety monitoring system, providing valuable reference for slope safety monitoring.

## 1. Introduction

The stability of mine slopes directly affects the safety, production, and ecological environment of mines [1]. As slope geometry and stress conditions continuously evolve, coupled with factors such as rainfall infiltration, weak structural planes, and complex geological formations, the risk of geological disasters such as mine slope instability significantly increases, posing a serious threat to people’s lives, property, and the environment [2,3]. Therefore, high-precision slope deformation monitoring and prediction are important research topics to ensure safe operation in mines [4,5,6].

At present, the research on slope deformation prediction mainly includes model-driven methods and data-driven methods [7,8]. The model-driven method establishes a geomechanical model or empirical statistical model to analyze the influence of relevant factors on deformation and makes predictions, such as expert empirical models and landslide mechanism models [9]. However, due to the complex geological conditions and susceptibility to the coupling effects of multiple factors, these methods are often limited in applicability and prediction accuracy. The data-driven method utilizes data mining techniques to process and analyze historical deformation monitoring data in order to predict surface displacement [10]. Given the coupled multi-factor influences and the nonlinear, dynamically evolving behavior of mine slopes [11]. With the development of deep learning, data-driven methods, such as Extreme Learning Machines (ELMs) [12], Support Vector Regression (SVR) [13], and Recurrent Neural Networks (RNNs) [14], have gradually gained people’s attention in the field of hazard monitoring due to their powerful feature extraction and nonlinear fitting capabilities.

The SVR model can effectively capture the complex nonlinear relationship between multiple factors and landslide displacement through a kernel function mapping the original feature space to a high-dimensional feature space. It can demonstrate good generalization capability even with limited monitoring data. The ELM model with minimal hyperparameter tuning can offer high training efficiency and is suitable for real-time slope displacement prediction. However, both SVR and ELM treat landslide displacement prediction as a static regression task, which limits their ability to capture the inherent dynamic characteristics in monitoring time series [15,16]. Considering the nonlinear and dynamic evolution characteristics of landslide displacement, many scholars are increasingly focusing on RNN, especially LSTM. The LSTM, with gate-controlled structure and memory cells, can capture long-term temporal dependencies in sequential data [17,18,19]. Studies showed that it had a higher prediction accuracy than traditional RNN, SVR, Back Propagation Neural Networks (BPNNs), and Support Vector Machines (SVMs) approaches in the displacement prediction for landslides and mine slopes [15,20,21]. Meanwhile, the CNN had also been applied to landslide displacement prediction due to its advantages in multi-modal monitoring data fusion and feature extraction. It can effectively learn the complex nonlinear relationships between internal and external factors in multi-modal data and accurately identify local deformation characteristics [18,22].

Although previous studies had shown the benefits of data-driven methods, single prediction models (such as LSTM, CNN, etc.), due to their simple structure, cannot fully extract the complex and nonlinear features in deformation monitoring data, which limited further improvements in prediction accuracy [23]. In contrast, hybrid modeling methods integrated the complementary advantages of multiple algorithms, thereby improving prediction performance [24]. Especially the CNN-LSTM model, benefiting from its synergistic capability of extracting spatial features and capturing temporal dependencies, was well-suited for processing landslide monitoring data with complex spatiotemporal characteristics and has demonstrated excellent predictive performance in this field [25,26,27]. These hybrid prediction models can improve the prediction accuracy of the model, but at the cost of requiring more hyperparameters. Some studies have explored hyperparameter optimization strategies based on intelligent optimization algorithms, including genetic algorithms [28], particle swarm optimization [29], and grey wolf optimization [30], etc. However, these algorithms were prone to local optima, slow convergence, and low computational efficiency [31].

Given the above, we constructed a Bayesian-optimized CNN-LSTM model for predicting mine slope displacement. This model effectively integrated the synergistic advantages of CNN and LSTM and introduced a Bayesian optimization algorithm to adaptively optimize the model hyperparameters, thereby constructing a fully slope displacement prediction framework. The study systematically evaluated the feasibility and effectiveness of this method in a typical mine slope displacement prediction and achieved more accurate displacement prediction based on multi-modal data, including GNSS displacement, rainfall, and earth pressure.

## 2. Materials and Methods

### 2.1. CNN-LSTM Model

**Figure 1:**
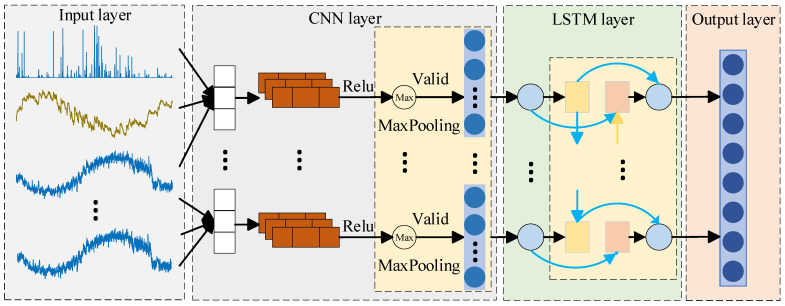
CNN-LSTM model network structure.

The CNN-LSTM model achieves feature extraction and temporal modeling of multi-modal time series data by concatenating CNN and LSTM. Its network structure is shown in Figure 1.

In the network structure of CNN-LSTM, the CNN is located at the front end of the network, which consists of an input layer, several hidden layers, and an output layer. The hidden layers include convolutional layers, activation functions (such as ReLU), and pooling layers. The convolutional layer, as the core component [32], performs convolution operations on multivariate time series, using Equation (1).

(1) $$Y(i,j)=\overset{M}{\underset{m=1}{\sum }}\overset{N}{\underset{m=1}{\sum }}W(i,j)*I(i+m,j+n)+b$$

where Y(i,j) denotes output feature data, W(i,j) represents convolutional kernel weights, ∗ signifies the convolution operation, I(i+m,j+n) indicates input data, and b is the bias term.

When predicting slope displacement, GNSS displacement, rainfall, and earth pressure are used as input variables for the CNN model. By stacking multiple convolutional layers, CNN can progressively extract deep features from raw monitoring data that reflect the evolutionary characteristics of slope displacement [33]. Then the pooling layer performs dimensionality reduction on the features through downsampling operations, thereby reducing computational complexity and enhancing the model’s adaptability to local perturbations and scale variations.

As the backend module of the CNN-LSTM model, LSTM is mainly used to model the high-dimensional features output by CNN in time series to reveal the long-term temporal dependence of the slope displacement evolution process. The LSTM networks are one of the most widely used types of RNNs [34]. By introducing memory units and gating mechanisms, it effectively overcomes the problems of gradient vanishing and exploding during training [35], enabling its extensive application in sequential prediction tasks. An LSTM unit consists of a forget gate, an input gate, and an output gate [36]. These gating units jointly regulate the retention and forgetting of information, consequently determining the update of the memory cell state. At each time step t, the hidden state $h_{t}$ of the LSTM is updated based on the current input feature $x_{t}$ and the previous state $h_{t-1}$, and the memory cell state $c_{t}$ is updated simultaneously. The calculation process is shown in Equation (2).

(2) $$\left\{\begin{array}{c} f_{t}=\sigma (W_{f}[h_{t-1},x_{t}]+b_{f})\\ i_{t}=\sigma (W_{i}[h_{t-1},x_{t}]+b_{i})\\ o_{t}=\sigma (W_{o}[h_{t-1},x_{t}]+b_{o})\\ c_{t}=c_{t-1}f_{t}+i_{t}\tanh(W_{c}[h_{t-1},x_{t}]+b_{c})\\ h_{t}=o_{t}\tan h(c_{t}) \end{array}\right.$$

where $f_{t}$, $i_{t}$, and $o_{t}$ denote the output values of the forget gate, input gate, and output gate, respectively; σ(⋅) and tanh(⋅) represents the sigmoid and hyperbolic tangent activation functions, respectively; W and b corresponds to the weight matrices and bias vectors of the respective gating units; $x_{t}$ is the feature vector at time step *t* output from the CNN pooling layer.

The last layer of the CNN-LSTM model is a fully connected layer, which is used to map the high-dimensional features output by the LSTM to the final displacement prediction results.

### 2.2. Bayesian Optimization Algorithms

Hyperparameter optimization is crucial for improving the predictive accuracy, generalization capability, and robustness of the model [37]. To improve the hyperparameter optimization strategy, this study introduced a Bayesian optimization algorithm to overcome issues such as hyperparameters becoming trapped in local optima, consequently enhancing the optimality of hyperparameter selection and the overall performance of the model. The core idea of the Bayesian optimization algorithm is to construct a posterior probabilistic model of the objective function and to intelligently select the next evaluation point by means of an acquisition function, therefore efficiently searching for the global optimum of a complex objective function [38]. The algorithm fully exploits information from historical observations of slope displacement, earth pressure, and rainfall, guiding the search process to converge toward regions of the potentially optimal solution. This optimization algorithm is typically implemented based on a Gaussian Process (GP). Given a set of previously selected points $X=\left[x_{1},x_{2},\ldots x_{n}\right]$ and their corresponding observations $Y=\left[y_{1},y_{2},\ldots y_{n}\right]$, the probabilistic distribution of the objective function f(x) is characterized by defining a mean function and a covariance function [39,40,41]. Specifically, $f(x)∼\mathrm{GP}(m(x),k(x,x_{i}))$ where m(x) and $k(x,x_{i})$ denote the mean function and the covariance function of the Gaussian Process, respectively, as shown in Equation (3).

(3) $$\left\{\begin{array}{l} m(x)=E[f(x)]\\ k(x,x_{i})=\sigma_{f}^{2}\exp[-\frac{1}{2}{\left(x-x_{i}\right)}^{T}(x-x_{i})] \end{array}\right.$$

where $\sigma_{f}^{2}$ is the signal variance, which is used to control the range of fluctuations of the function values.

The posterior probability distribution of the objective function f(x) can be obtained through the Gaussian process model. In this study, the objective function is defined as the RMSE of the model under hyperparameter x, and minimizing the RMSE is taken as the optimization objective. In order to select the next optimal sampling point, the expected improvement (EI) is used as the acquisition function. By selecting the parameter point with the largest EI value as the next evaluation point, the optimal solution of the objective function can be efficiently approximated, as shown in Equation (4).

(4) $$\mathrm{EI}(x)=E(\max(y^{*}-y,0))$$

where $y^{*}$ denotes the best function value observed so far, i.e., the optimal value among the evaluated points, and y represents a possible value of the objective function at point x.

### 2.3. CNN-LSTM Model Based on Bayes Optimization

**Figure 2:**
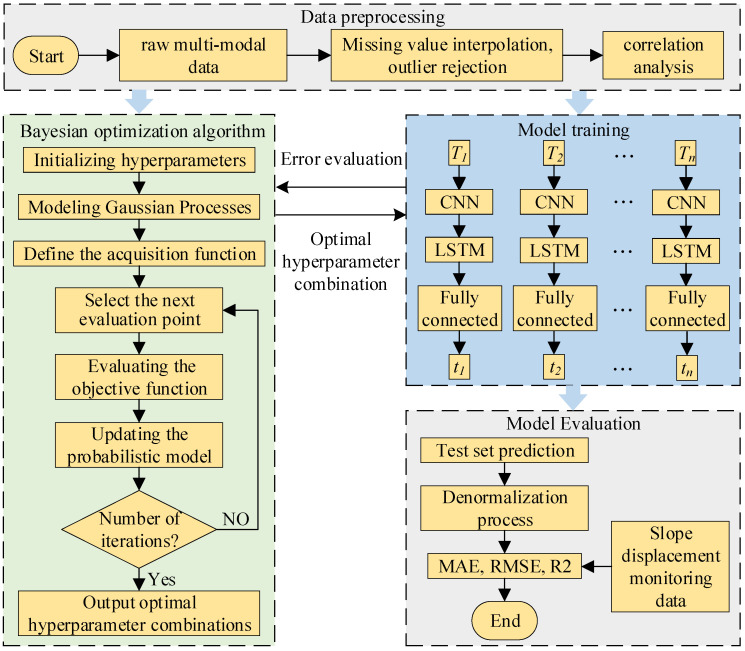
Flowchart of Bayesian optimization of CNN-LSTM model.

Bayesian optimization was employed to tune the hyperparameters of the CNN-LSTM model, in which three key hyperparameters, namely the number of hidden units (NumOfUnits), the initial learning rate (InitialLearnRate), and the L2 regularization coefficient (L2Regularization), are adaptively optimized. The model’s workflow is shown in Figure 2.

Firstly, target monitoring points were selected, and the raw multi-modal monitoring data were collected. Cubic spline interpolation and the Pauta criterion (3σ rule) were then applied to preprocess the raw data. The processed data samples were subsequently divided into a training set and a test set.

Secondly, the range of hyperparameter values for the CNN-LSTM model was defined. Then, the optimal combination of hyperparameters was optimized using a Bayesian optimization algorithm, which was saved and subsequently used to train the CNN-LSTM model.

Finally, the test set was fed into the CNN-LSTM model to generate predictions. The predicted results were then denormalized to obtain the final slope displacement prediction values. Subsequently, the predictions were compared with displacement reference values, and the model performance was evaluated using relevant metrics such as MAE, RMSE, and R^2^.

### 2.4. Model Evaluation Metrics

In this study, three metrics were selected to evaluate the prediction model performance: mean absolute error (MAE), root mean squared error (RMSE), and coefficient of determination (R^2^) [42]. The corresponding calculation formulas were presented in Equation (5):

(5) $$\left\{\begin{array}{c} \mathrm{MAE}=\frac{1}{n}\sum_{i=1}^{n}|T_{i}-t_{i}|\\ \mathrm{RMSE}=\sqrt{\frac{1}{n}\sum_{i=1}^{n}(T_{i}-t_{i}{)}^{2}}\\ R^{2}=1-\frac{\sum_{i=1}^{n}(T_{i}-t_{i}{)}^{2}}{\sum_{i=1}^{n}(T_{i}-\bar{T}{)}^{2}} \end{array}\right.$$

where $T_{i}$ and $t_{i}$ denote the reference and predicted values of slope displacement, respectively; $\bar{T}$ represents the mean reference value of the slope displacement; and n is the number of samples.

Among them, smaller values of MAE and RMSE indicate better model performance. R^2^ is primarily used to assess the goodness of fit of the prediction model, and a value closer to 1 implies superior predictive performance.

## 3. Experimental Analysis

### 3.1. Data Sources

The monitored slope is located in the GuShan open-pit iron mine, and the studied area is a post-mining bench slope. The area is located in a subtropical monsoon climate zone, characterized by distinct seasons, abundant sunshine, ample rainfall, and the simultaneous occurrence of high temperatures and rainfall. The slope monitoring area extends approximately 1100 m in the east-west direction and about 1000 m in the north-south direction, with the lowest elevation of −180 m at the slope toe. Geometrically, the slope is composed of 17 bench levels, each with an average height of approximately 12 m, and the slope angle ranges from 35° to 45°. The large slope angles combined with the multi-level bench structure result in a high susceptibility to geological hazards, such as landslides.

From an engineering-geological perspective, the slope comprises an upper Quaternary cover overlying a bedrock slope. The Quaternary deposits mainly include water-bearing gravel-cobble layers, interbedded with silty clay and fine sand strata. The bedrock is dominated by andesite, volcanic breccia, and tuff; the upper benches are commonly fractured and strongly weathered, exhibiting scattered-fragmented to fractured blocky structures that reduce the overall strength of the rock mass. In addition, groundwater is present as pore water, confined pore water within gravel–cobble layers, and bedrock fissure water. These groundwater components can couple with rainfall infiltration, thereby altering internal stress states, which are relevant to subsequent deformation responses.

**Figure 3:**
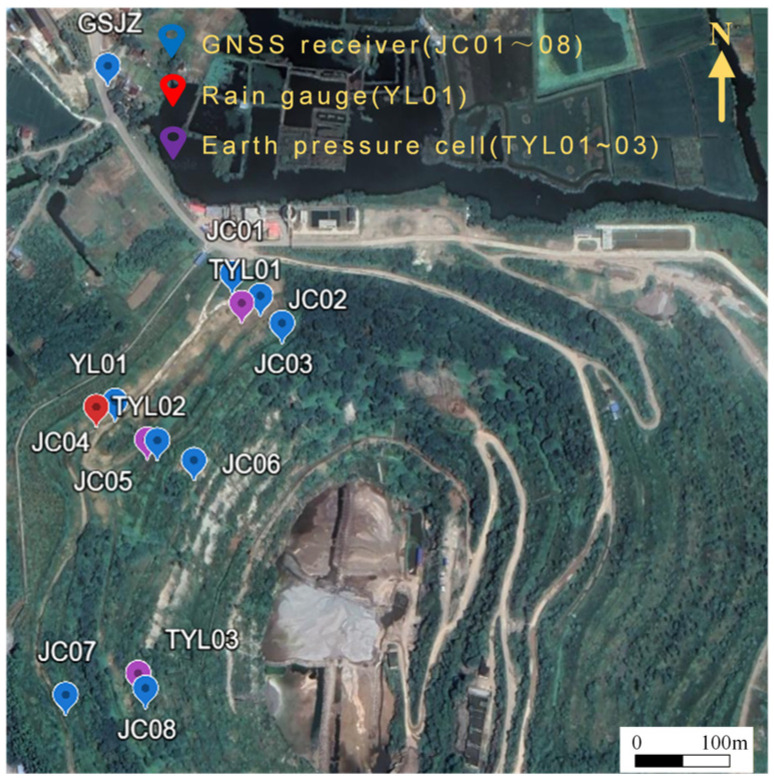
Spatial distribution of the monitoring sensors.

In this study, the monitoring data were collected long-term after mining ceased (The GuShan mine was operated from 1954 to 2014). Therefore, the primary external forcing considered in the prediction task is rainfall, together with the associated stress response reflected by earth pressure measurements. To acquire multi-modal monitoring data, we deployed one GNSS reference station (GSJZ), eight GNSS monitoring stations (JC01~JC08), one rain gauge (YL01), and three earth pressure gauges (TYL01~TYL03). The spatial distribution of these sensors was shown in Figure 3, and the parameters of the related instruments were listed in Table 1.

**Figure 4:**
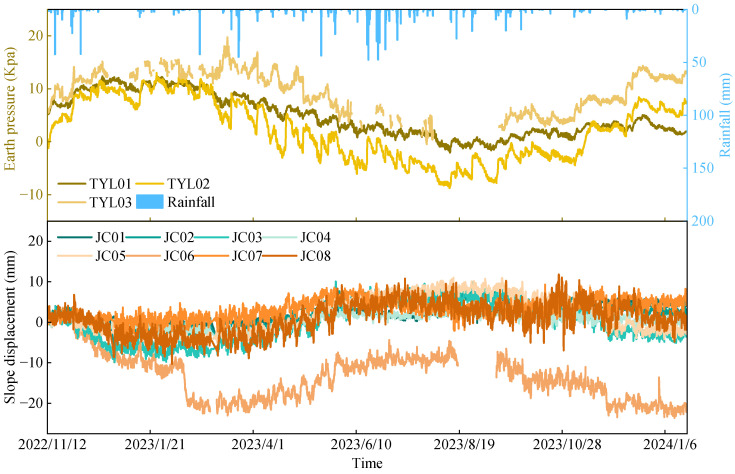
Monitoring information on slope displacement, rainfall, and earth pressure.

To enhance the ability of monitoring data to characterize the influence of slope displacement, multi-modal monitoring data were sampled at a uniform interval of 3 h, covering the period from 12 November 2022, to 20 January 2024. To ensure the continuity and reliability of the time series analysis, the missing data were completed using cubic spline interpolation, and outliers in the monitoring time series were identified and removed based on the Pauta criterion (3σ rule). Figure 4 shows the monitoring results of surface displacement, rainfall [43,44], and earth pressure [45,46] for the slope. As can be observed from the figure, the slope displacement has some small-amplitude fluctuations, with an overall variation ranging from −10 mm to 10 mm, forming an inverted “S”-shaped fluctuation pattern. The overall variation in earth pressure is relatively small, mostly concentrated between −10 kPa and 13 kPa. Rainfall shows pronounced spatial and temporal unevenness, with clear seasonal variability [47]. Among them, rainfall is usually one of the important external factors that induce and accelerate slope displacement. Rainfall infiltration leads to a decrease in matric suction and an increase in the bulk density of the slope body, resulting in a decrease in shear strength and an increase in sliding force, thus affecting slope stability. Meanwhile, earth pressure [48,49] can serve as one of the representative variables of the internal stress state and stress adjustment process of the slope body, and its changes can reflect the deformation trend and stability of the slope.

Considering the differences in deformation characteristics, stress conditions, and multi-modal monitoring data among various locations of the slope, monitoring points JC03 and JC05, located on platforms at different elevations, were selected as the study objects. These two points were chosen to capture both the overall deformation trend of the slope and the localized differential settlement characteristics. The remaining monitoring points were excluded from the analysis due to issues such as data loss, equipment failure, or spatial redundancy.

### 3.2. Correlation Analysis

**Figure 5:**
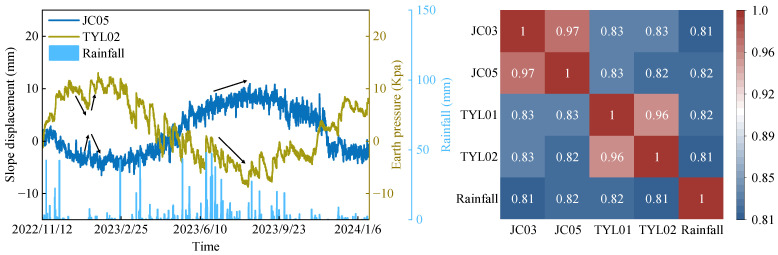
Data sequences (**left**) and grey relational degree (**right**) for slope displacement, rainfall, and earth pressure.

Grey relational analysis is a correlation analysis method based on grey system theory [50], commonly used to quantify the interaction and influence between two data sequences. Generally, if the grey relational degree is greater than 0.6, it is considered that the two have a high correlation [51]. The multi-modal dataset used in this study included five variables. The displacement data from monitoring points JC05 and JC03 were the dependent variables, while the other three variables (rainfall, TYL01, and TYL02) were considered independent variables. Figure 5 shows the data sequences of slope displacement, rainfall, and earth pressure, as well as the grey relational coefficients between them. Taking monitoring point JC05 as an example, its grey relational degree with both rainfall and TYL02 was 0.82. As shown in the figure, the overall trends in the amplitude and periodicity between slope displacement and earth pressure were consistent. For example, around 12 January 2023, a decrease in earth pressure corresponded to a reduction in the magnitude of slope displacement, and vice versa. This phenomenon can be attributed to the fact that an increase in earth pressure led to higher shear stress within the slope, thereby resulting in greater displacement. Rainfall led to more pronounced abrupt changes in slope displacement. In addition, Figure 5 shows that the grey relational degrees among all sets of monitoring data exceed 0.6, further confirming that slope displacement variations are strongly influenced by rainfall and earth pressure and exhibit strong correlations.

### 3.3. Hyperparameter Optimization and Model Training

The training sets and testing sets were constructed by using 70% and 30% of the multi-modal monitoring dataset, respectively. Specifically, 2456 sets of monitoring data from 00:00 on 12 November 2022 to 21:00 on 14 September 2023 were used as the training sets for model training, while 1024 sets of monitoring data from 00:00 on 15 September 2023 to 21:00 on 20 January 2024 were used as testing sets for performance evaluation. The parameter settings of the Bayes-CNN-LSTM model are shown in Table 2.

Taking the slope displacement data from the monitoring point JC05 as an example, Table 3 presents the optimization process of the key model hyperparameters. The Bayes-CNN-LSTM model employed a sliding window mechanism for prediction. Based on multiple pre-experiments, the previous 20-step displacement sequence (lagged monitoring samples) and the rainfall and earth pressure values at the prediction time step were used as input to predict the displacement at the corresponding time step. The key hyperparameters of the model were adaptively searched within the parameter space using the Bayesian optimization algorithm. As shown in Table 3, the optimization process was carried out for ten iterations, at which point the objective function (RMSE) showed a convergence trend. The objective function value generally decreased with the increase in the number of iterations. Among them, the first iteration performed well due to the relatively reasonable initial parameter settings, but the fifth iteration obtained the smallest RMSE value, indicating that the model performance reached an optimum in this search. Therefore, the hyperparameter combination obtained in the fifth iteration was selected as the optimal configuration of the model, with the NumOfUnits, InitialLearnRate, and L2Regularization being 27, 0.011142, and 2.383 × 10^−7^, respectively.

### 3.4. Prediction Results and Accuracy Analysis

**Figure 6:**
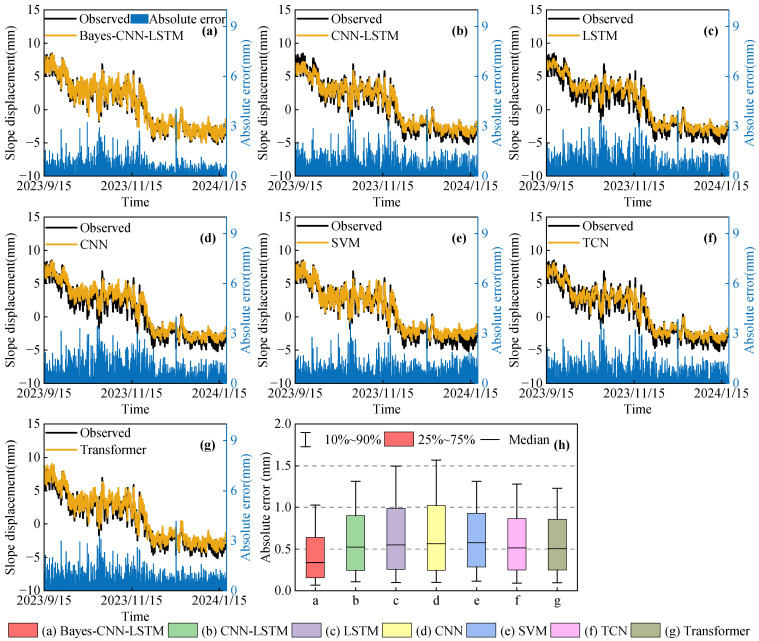
Displacement predictions and absolute error analysis for different models at monitoring point JC03: (**a**) Bayes-CNN-LSTM, (**b**) CNN-LSTM, (**c**) LSTM, (**d**) CNN, (**e**) SVM, (**f**) TCN, (**g**) Transformer; (**h**) absolute error distributions for all models.

**Figure 7:**
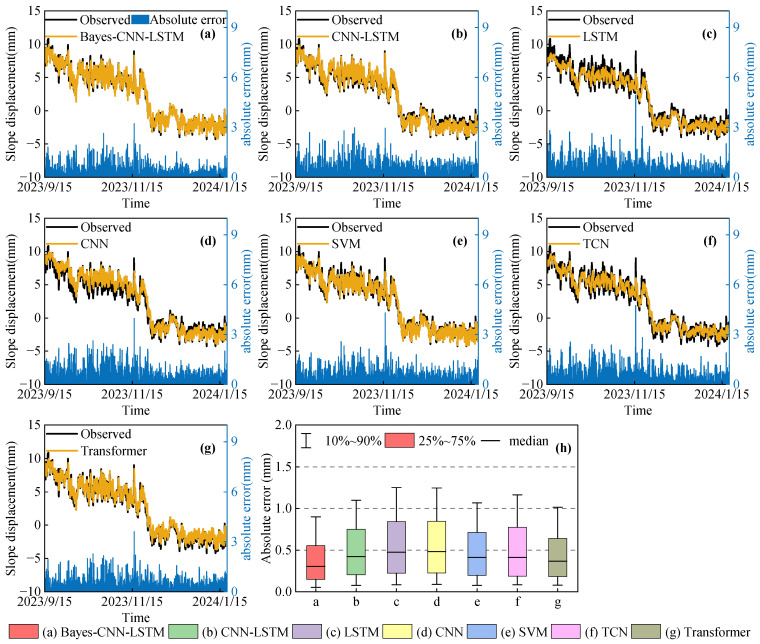
Displacement predictions and absolute error analysis for different models at monitoring point JC05: (**a**) Bayes-CNN-LSTM, (**b**) CNN-LSTM, (**c**) LSTM, (**d**) CNN, (**e**) SVM, (**f**) TCN, (**g**) Transformer; (**h**) absolute error distributions for all models.

To verify the superiority of the constructed Bayes-CNN-LSTM model in terms of prediction accuracy, several typical prediction methods, including CNN-LSTM [52], LSTM [53], CNN [54], SVM [55], TCN [56], and Transformer [57], were selected for comparison. Slope displacement of monitoring points JC03 and JC05 was predicted and analyzed. The prediction values and the absolute prediction errors of each model are illustrated in Figure 6 and Figure 7. To quantitatively evaluate the predictive performance of different models, Table 4 and Table 5 show the MAE, RMSE, and R^2^ evaluation indicators of each model’s predictions at the two monitoring points.

As can be observed from Figure 6 and Figure 7, the prediction values of all models generally followed the actual displacement trend. However, compared with the CNN-LSTM, LSTM, CNN, SVM, TCN, and Transformer models, the Bayes-CNN-LSTM model exhibited the best fitting performance in the slope displacement evolution process, indicating that this model can effectively capture the main variation characteristics of slope displacement.

Figure 6h and Figure 7h show the distributions of the absolute prediction errors of different models at monitoring points JC03 and JC05. The results indicated that the Bayes-CNN-LSTM model yielded the smallest overall prediction errors at both monitoring points. Specifically, at monitoring point JC03, more than 90% of the absolute prediction errors were less than 1.1 mm, and approximately 75% were less than 0.7 mm. At monitoring point JC05, over 90% of the errors were within 1.0 mm, with about 75% being less than 0.6 mm. Overall, these results demonstrated that the Bayes-CNN-LSTM model was capable of accurately learning the local fluctuation characteristics during slope displacement evolution, maintaining small and stable prediction errors in most cases, and exhibiting excellent predictive performance.

As indicated by Table 4 and Table 5, the conventional baselines (LSTM, CNN, and SVM) exhibited a certain level of predictive capability, but there was still room for improvement. Compared with these models, the CNN-LSTM model enhanced prediction performance, demonstrating that when faced with complex input features, CNN-LSTM can achieve collaborative modeling in both space and time, enabling more effective learning and training. In addition, we observed that the Transformer-based model delivers consistently strong and stable performance across monitoring points, whereas the TCN baseline is also competitive but exhibits point-dependent variability. By incorporating the Bayesian optimization algorithm, globally optimal hyperparameters can be identified, which further improved the prediction accuracy of the Bayes-CNN-LSTM model. For instance, at point JC03, the MAE, RMSE, and R^2^ values reached 0.470 mm, 0.660 mm, and 0.964, respectively; at point JC05, the MAE, RMSE, and R^2^ values were 0.417 mm, 0.576 mm, and 0.978, respectively. In summary, at point JC03, the MAE of the Bayes-CNN-LSTM model was decreased by 27.9%, 32.8%, 34.2%, 30.0%, 25.3%, and 23.5% compared with the CNN-LSTM, LSTM, CNN, SVM, TCN, and Transformer models, while the RMSE was decreased by 22.1%, 27.9%, 28.9%, 22.0%, 19.8%, and 17.0%, respectively. At point JC05, the MAE was decreased by 22.2%, 29.7%, 30.4%, 18.2%, 24.0%, and 11.8%, and the RMSE was decreased by 18.1%, 25.8%, 30.0%, 13.9%, 21.5%, and 7.8%, respectively. These results indicated that the Bayes-CNN-LSTM model can effectively capture slope displacement characteristics at different monitoring points, exhibiting superior prediction accuracy and stability.

### 3.5. Extrapolation Prediction Results and Analysis

Currently, displacement information remains the primary data source for slope monitoring. However, in practical engineering applications, GNSS displacement observations are often affected by certain factors such as equipment failures, signal obstruction, and complex observation environments, which can lead to some problems such as data loss, degraded data quality, and even monitoring failure. Meanwhile, extrapolative prediction of slope displacement has more important practical significance for disaster prevention and reduction. Therefore, two experiments were used to analyze the extrapolation prediction capability of the Bayes-CNN-LSTM model when GNSS is unavailable. In Experiment I, primarily analyzed the contribution of different external influencing factors to the extrapolation prediction based on the Bayesian-CNN-LSTM model, while Experiment II analyzed the predictive performance of the Bayes-CNN-LSTM model and other typical models. Both experiments were conducted based on trained models, using the same data with an interval of 3-h to predict slope displacement over the subsequent 24-h (8 steps) and 48 h (16 steps).

**Figure 8:**
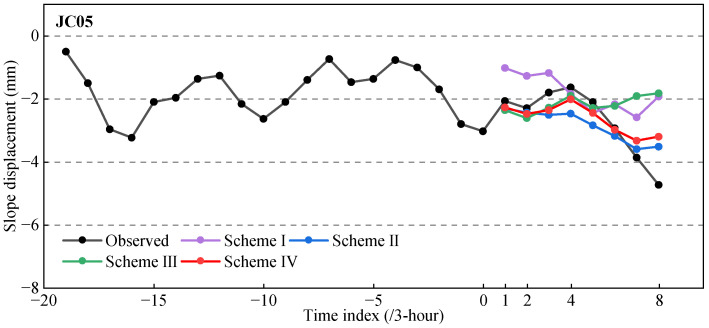
Extrapolation prediction results of slope displacement under different schemes.

**Figure 9:**
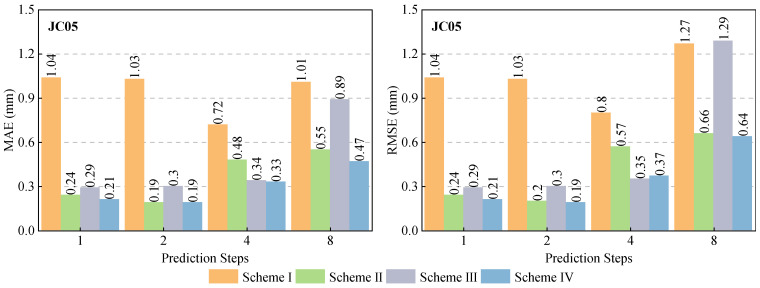
Prediction accuracy analysis of various schemes under different prediction step lengths.

In Experiment I, we analyzed the contribution of different external influencing factors (rainfall and earth pressure) to slope displacement prediction by controlling the input feature set of the Bayes-CNN-LSTM model. Specifically, four input configurations were designed: (1) Scheme I used only the lagged displacement sequence as input; (2) Scheme II introduced rainfall data based on the lagged displacement sequence; (3) Scheme III introduced earth pressure data based on the lagged displacement sequence; (4) Scheme IV integrated the lagged displacement sequence, rainfall data, and earth pressure data simultaneously. Here, the lagged displacement sequence refers to the previous 20 samples with a 3-h interval (i.e., a 60-h time window), covering the period from 12:00 on 18 January 2024 to 21:00 on 20 January 2024. The results were shown in Figure 8 and Figure 9.

As can be seen from Figure 8, Scheme III, by introducing earth pressure data, significantly improved the model’s accuracy in predicting peak displacement and inflection points, with its predictions more closely matching the actual reference values at these key locations. After using rainfall data, Scheme II effectively enhanced the model’s ability to capture the overall slope displacement trend, with its predictions more closely resembling the actual reference values compared to Scheme III. Although introducing only one external factor (rainfall or earth pressure) in schemes II and III can improve model performance to some extent, the prediction error still shows significant fluctuations. Due to the comprehensive consideration of the effects of rainfall and earth pressure, Scheme IV got the best balance between trend fitting and detail capture, thus having the highest overall prediction accuracy.

The MAE and RMSE values of four schemes are shown as in Figure 9. Overall, compared with Schemes II and III, the MAE of Scheme IV was decreased by 14.5% and 47.1%, respectively, and RMSE decreased by 0.3% and 50.4%, respectively. And compared with Scheme I, MAE and RMSE were decreased by 53.5% and 49.6%, respectively. This fully demonstrated that external influencing factors played a crucial role in slope displacement prediction and that the reasonable fusion of rainfall and earth pressure information can improve the model’s extrapolation prediction performance and robustness.

**Figure 10:**
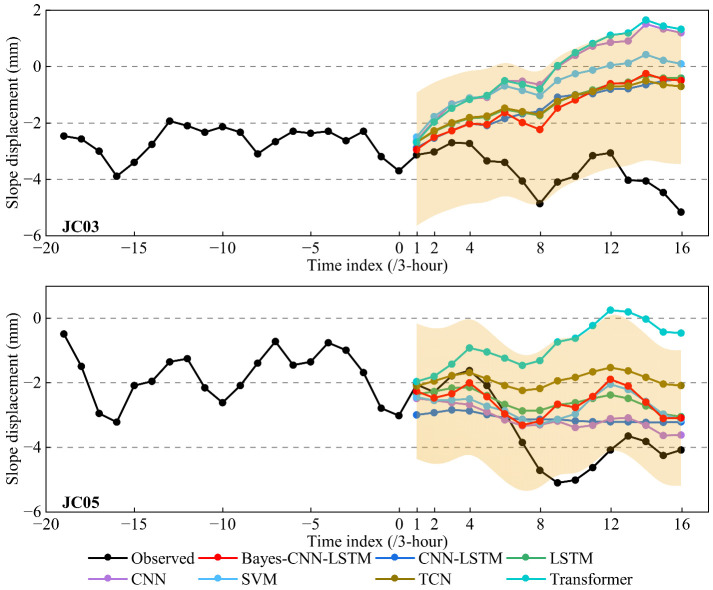
Extrapolation predictions of slope displacement from each model.

**Figure 11:**
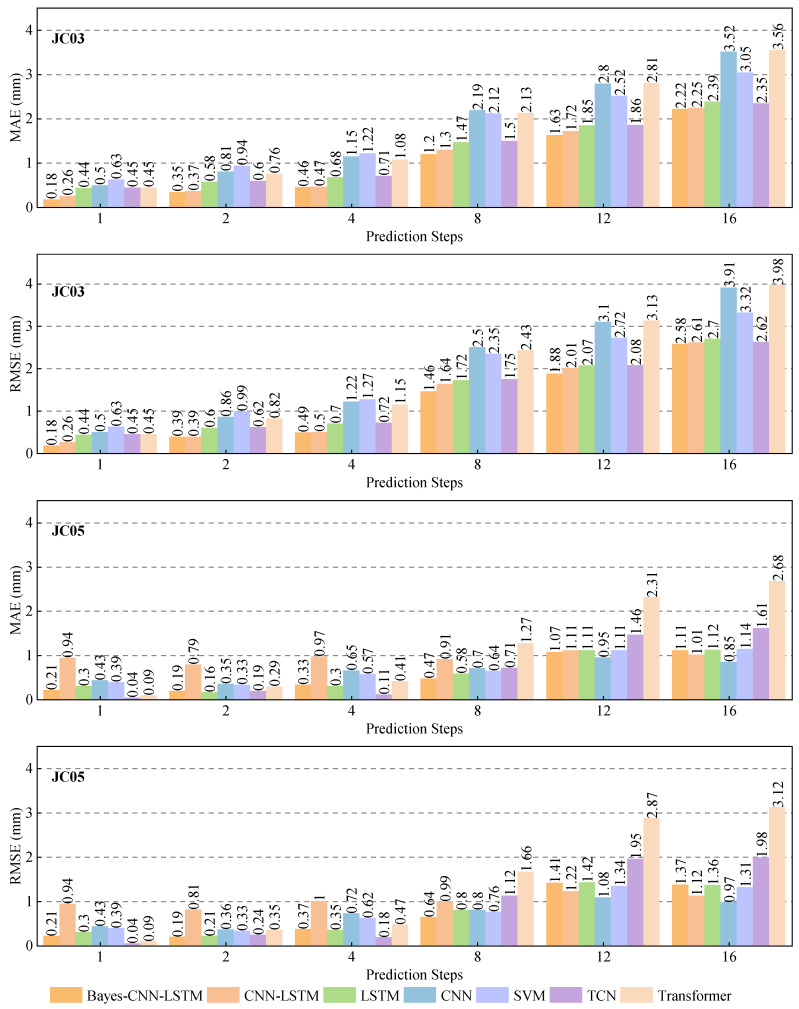
Extrapolation accuracy of each model at different prediction horizons.

Based on the above conclusions, Experiment II was conducted using lagged displacement sequence, rainfall data, and earth pressure data as inputs to further analyze the extrapolation performance of the Bayes-CNN-LSTM model and other typical models. The extrapolation prediction results are presented in Figure 10 and Figure 11.

As shown in Figure 10, the prediction accuracy of all models decreased with increasing extrapolation time and accumulating errors. The prediction error from the previous time step was propagated and accumulated as input for subsequent steps; therefore, the longer the prediction horizon, the more likely the uncertainty and bias will increase. However, the Bayes-CNN-LSTM model’s prediction results (red curve) still had relatively smaller prediction errors. Furthermore, it maintained high consistency with the displacement reference values in both long-term trends and short-term fluctuations, demonstrating excellent feature-capturing capability, especially at detailed points such as displacement turning points. In contrast, the prediction results output by some comparative models were smoother, with smaller fluctuations, and exhibited a certain degree of “under-sensitivity” to actual displacement changes. The shaded region in Figure 10 represented the 95% confidence interval of predictions from the Bayes-CNN-LSTM model constructed by quantile regression, which was used to characterize the uncertainty of the model’s prediction results.

Figure 11 shows the comparison of MAE and RMSE for each model with step lengths of 1 (3-h), 2 (6-h), 4 (12-h), 8 (24-h), 12 (36-h), and 16 (48-h). Overall, the Bayes-CNN-LSTM model demonstrated stable performance in short-to-medium-term extrapolation, especially at point JC03, achieving superior error levels across different step lengths. For example, when the extrapolation prediction step length was 8 (24-h), the MAE and RMSE of monitoring point JC03 were 1.20 mm and 1.46 mm. Compared with the CNN-LSTM, LSTM, CNN, SVM, TCN, and Transformer models, the MAE was decreased by 7.7%, 18.4%, 45.2%, 43.4%, 20.0%, and 43.7%, while the RMSE was decreased by 11.0%, 15.1%, 41.6%, 37.9%, 16.6%, and 39.9%, respectively. When the step length was further extended to 16 (48-h), the MAE and RMSE remained at 2.22 mm and 2.58 mm, respectively, indicating that the overall error growth was controllable and the trend tracking ability was strong. At point JC05, with a step length of 8 (24-h), the MAE and RMSE were 0.47 mm and 0.64 mm, decreased by 48.4%, 19.0%, 32.9%, 26.6%, 33.8%, and 63.0% in MAE and 35.4%, 20.0%, 20.0%, 15.8%, 42.9%, and 61.4% in RMSE compared with the other models, respectively. When the step length was 16 (48-h), some baseline models showed locally smaller values in MAE and RMSE, but as seen in the extrapolation curves in Figure 10, their output tended to be smoother, failing to adequately respond to local fluctuations and turning points. In contrast, Bayes-CNN-LSTM maintained better trend tracking and fluctuation characterization capabilities within the 36–48 h prediction range.

In brief, rainfall and earth pressure information both played a certain role in displacement prediction, and considering their combined effects can improve the sensitivity and adaptability of the model to complex nonlinear changes in slope displacement. And the Bayes-CNN-LSTM model maintained relatively strong stability and predictive capability under a GNSS-unavailable environment, enhancing the applicability of slope safety monitoring systems.

## 4. Conclusions

To improve the accuracy and stability of slope displacement prediction, a Bayesian-optimized CNN-LSTM prediction model is constructed in this study. This model effectively integrates the advantages of CNN and LSTM and introduces a Bayesian optimization algorithm to adaptively optimize the model hyperparameters. We analyze the predictive performance of the constructed model using multi-modal monitoring data from the Gushan slope, and the main conclusions are summarized as follows:

(1) The Bayesian-optimized CNN-LSTM model easily avoids the problem of hyperparameters becoming trapped in local optima. Experimental results show that this model performs well in terms of both prediction accuracy and fitting effect and can effectively predict slope displacement.
(2) Compared with other mainstream models, the constructed Bayes-CNN-LSTM model shows higher prediction accuracy. At monitoring point JC03, the MAE and RMSE are 0.470 mm and 0.660 mm, respectively, and the average decreased by 29.0% and 23.0% compared with the comparison models. At point JC05, the MAE and RMSE are 0.417 mm and 0.576 mm, respectively, and the average decreased by 22.7% and 19.5%.
(3) Regarding the influence of external factors on slope displacement prediction, experimental results show that the MAE and RMSE of the model predictions are 0.47 mm and 0.64 mm when using both rainfall and earth pressure, respectively. Therefore, the proper integration of multi-modal data can effectively improve the performance of slope displacement prediction models.
(4) The Bayes-CNN-LSTM model exhibits good extrapolation capability, demonstrating better prediction accuracy and stability even at longer prediction step lengths. For example, in the prediction step length of 8 (24-h), the MAE and RMSE at monitoring points JC05 are decreased by 30.2% and 24.6%, respectively. Future work will focus on collecting data from multiple slopes for generalized validation and simultaneously explore the integration of transfer learning and physical priors to improve applicability across different scenarios.

## Figures and Tables

**Table 1:** Description of monitoring sensor equipment.

Serial Number	Sensor Type	Measurement Data	Monitoring Point
1	GNSS receiver	surface displacement	JC01~JC08
2	Rain gauge	quantity of rainfall	YL01
3	Earth pressure cell	earth pressure	TYL01~TYL03

**Table 2:** Parameter configuration of the Bayes-CNN-LSTM Model.

Category	Parameter	Value or Search Space
Hyperparameter Optimization	NumOfUnits	[10, 200]
	InitialLearnRate	[1 × 10^−5^, 0.1]
	L2Regularization	[1 × 10^−10^, 0.01]
CNN Architecture	Convolutional kernel size	10
	Convolutional layer 1	32
	Convolutional layer 2	64
	Pooling stride	10
	Activation function	ReLU
LSTM Architecture	LSTM unit	50
	Dropout Rate	0.25
Training Configuration	Optimizer	Adam
	Max Epochs	500
	Learn Rate Drop Period	400
	Learn Rate Drop Factor	0.2
Bayesian Optimization	Max Evaluations	10

**Table 3:** Hyperparameter optimization process of the Bayes-CNN-LSTM.

Iter	Eval Result	Objective	Runtime (s)	BestSoFar (Observed)	BestSoFar (Estim.)	NumOfUnits	InitialLearnRate	L2Regularization
1	Best	0.5335	7.8	0.5335	0.5335	123	0.0021821	5.9802 × 10^−9^
2	Accept	0.54125	6.6	0.5335	0.53414	175	0.059871	2.2674 × 10^−5^
3	Accept	0.84648	7.0	0.5335	0.53352	190	1.6551 × 10^−5^	9.2554 × 10^−8^
4	Accept	0.58802	6.8	0.5335	0.53351	45	0.00022002	0.00016301
**5**	**Best**	**0.415**	**6.7**	**0.415**	**0.41503**	**27**	**0.011142**	**2.383** **× 10** **^−^** **^7^**
6	Accept	0.47323	6.7	0.415	0.41507	12	0.095701	1.3512 × 10^−9^
7	Accept	0.45736	6.7	0.415	0.43897	10	0.0048592	1.5851 × 10^−8^
8	Accept	0.48132	6.6	0.415	0.451	10	0.012249	1.8481 × 10^−6^
9	Accept	0.57964	6.6	0.415	0.41508	10	0.015724	0.00066291
10	Accept	0.44038	7.2	0.415	0.43268	66	0.030152	1.2545 × 10^−8^

Bold indicates the iteration with the minimum RMSE (selected optimal hyperparameter configuration).

**Table 4:** Comparison of prediction performance of different models at monitoring point JC03 (MAE, RMSE, and R^2^).

Model	MAE (mm)	RMSE (mm)	R^2^
Bayes-CNN-LSTM	**0.470**	**0.660**	**0.964**
CNN-LSTM	0.652	0.847	0.941
LSTM	0.699	0.916	0.931
CNN	0.714	0.928	0.928
SVM	0.671	0.846	0.941
TCN	0.629	0.823	0.944
Transformer	0.614	0.795	0.949

Bold indicates the best results.

**Table 5:** Comparison of prediction performance of different models at monitoring point JC05 (MAE, RMSE, and R^2^).

Model	MAE (mm)	RMSE (mm)	R^2^
Bayes-CNN-LSTM	**0.417**	**0.576**	**0.978**
CNN-LSTM	0.536	0.703	0.968
LSTM	0.593	0.776	0.961
CNN	0.599	0.778	0.961
SVM	0.510	0.669	0.971
TCN	0.549	0.734	0.965
Transformer	0.473	0.625	0.974

Bold indicates the best results.

## Data Availability

The multi-modal monitoring data of mine slopes used in this study will not be disclosed due to confidentiality agreements signed with collaborating units and related data security policies.
